# Cell proliferation is related to *in vitro* drug resistance in childhood acute leukaemia

**DOI:** 10.1038/sj.bjc.6600787

**Published:** 2003-03-04

**Authors:** P Kaaijk, G J L Kaspers, E R van Wering, G J Broekema, A H Loonen, K Hählen, K Schmiegelow, G E Janka-Schaub, G Henze, U Creutzig, A J P Veerman

**Affiliations:** 1 Department of Pediatric Hematology/Oncology, VU University Medical Center, 9D PO Box 7057, 1007 MB Amsterdam, The Netherlands; 2 Dutch Childhood Leukemia Study Group, PO Box 43515, 2504 AM The Hague, The Netherlands; 3 Division of Oncology/Hematology, Sophia Children's Hospital, Dr Molewaterplein 60, 3015 GJ Rotterdam, The Netherlands; 4 University Hospital Rigshospitalet, Pediatric Clinic II, Blegdamsvej 9, 2100 Copenhagen, Denmark; 5 The COALL Study Group, Department of Hematology/Oncology, University Hospital Eppendorf, Martinistrasse 52, 20246 Hamburg, Germany; 6 The BFM-ALL relapse (REZ) Study Group, Department of Pediatric Hematology/Oncology, University Medical Center Charite, Augustenburger Platz 1, 13353 Berlin, Germany; 7 The AML-BFM Study Group, University Children's Hospital Münster, Albert Schweitzerstrasse 33, 48129 Münster, Germany

**Keywords:** childhood leukaemia, cell proliferation, drug resistance

## Abstract

Bone marrow and peripheral blood samples from 362 patients with acute lymphoblastic leukaemia (ALL) proliferating cell and 90 patients with acute myeloid leukaemia (AML) were analysed for S-phase fractions, Ki67 antigen, and proliferating cell nuclear antigen expression. The S-phase fractions were correlated with *in vitro* drug resistance to 15 different anticancer agents. Leukaemia cells isolated from bone marrow had higher S-phase fractions than leukaemia cells isolated from peripheral blood (in initial ALL, median values resp. 6.9 and 2.7%, in initial AML resp. 5.3 and 1.3%; both *P*<0.01). Relapse ALL samples derived from bone marrow showed increased S-phase fractions (median 9.9%) compared with initial ALL samples (median 6.9%; *P*<0.01). ALL samples obtained at initial diagnosis showed higher S-phase fractions (median 6.9%) and higher Ki67 expression (median 30%) than initial AML samples (median resp. 5.3 and 14%; both *P*<0.05). The S-phase fractions were not related to white blood cell count, age, or gender. Within initial ALL, the S-phase fraction correlated significantly but modestly strong (*ρ*=0.3–0.5; *P*<0.05) with sensitivity to antimetabolites (cytarabine, mercaptopurine, thioguanine), L-asparaginase, teniposide, and vincristine. Similar results were found within subgroups of initial ALL (nonhyperdiploid and common/precursor-B-lineage ALL). In relapsed ALL and AML such correlations were not found. In conclusion, cell proliferation differs between leukaemia subgroups and increased proliferation is associated with increased *in vitro* sensitivity to several anticancer agents in initial ALL.

The prognosis of childhood acute leukaemia has improved during the past decades. This has been accomplished by the introduction of refined schedules of combination chemotherapy (Reiter *et al*, 1994; Pui and Evans, 1998; Stevens *et al*, 1998; Creutzig *et al*, 2001). As a consequence of the improved prognosis and risk-adapted treatment, the prognostic significance of a number of parameters at presentation has decreased. At present, the most important prognostic features are age at presentation, white blood cell count, cell morphology, immunophenotype, and genotype (DNA ploidy and certain chromosomal abnormalities) (Creutzig *et al*, 1990; Kersey, 1997; Pui and Evans, 1999). More recently, *in vitro* drug resistance (Klumper *et al*, 1995b; Hongo *et al*, 1997; Kaspers *et al*, 1997,^1998^; Asselin *et al*, 1999) and the presence of minimal residual disease (Van Dongen *et al*, 1998; Eckert *et al*, 2001; Nyvold *et al*, 2002) in the course of treatment have shown to be strong independent prognostic factors in childhood acute lymphoblastic leukaemia (ALL).

Contradictory findings have been reported concerning the prognostic value of the S-phase fractions of leukaemia cells. In childhood acute leukaemia, the S-phase fraction has been found to correlate with clinical (prognosis and age) and cell biological features (immunophenotype and genotype) at presentation (Dow *et al*, 1982; Look *et al*, 1982; Smets *et al*, 1983,^1995^; Duque *et al*, 1993; Vidriales *et al*, 1995; Kamikubo *et al*, 2000; Braess *et al*, 2001). A review of the literature stated, however, that the S-phase fractions were not prognostic for treatment response or response duration in acute leukaemia in general (Duque *et al*, 1993). Nevertheless, others reported that bone marrow S-phase fractions of >6% were shown to be strongly predictive for poor outcome in childhood ALL patients (Dow *et al*, 1982; Smets *et al*, 1995). In agreement with these findings, in 204 acute myeloblastic leukaemia (AML) patients, a high number of cells in S phase related to shorter survival (Vidriales *et al*, 1995) and a low proportion of G_0_-phase cells during induction chemotherapy correlated with subsequent remission in AML (Kamikubo *et al*, 2000). In contrast, however, in a recent study with 187 AML samples that were cytogenetically classified into prognostic subgroups, a high proliferative activity was associated with a higher complete remission rate in both the normal (*n*=99) and unfavourable groups (*n*=29) (Braess *et al*, 2001).

It has been demonstrated by others that stimulation of cell proliferation of leukaemia cells by preincubation with growth factors could enhance *in vitro* sensitivity to cytarabine (Butturini *et al*, 1990; Bai *et al*, 1999). This indicates that pretreatment with growth factors as chemosensitising approach may be useful for patients with childhood acute leukaemia. In the present study, it was investigated if cell proliferation of acute childhood leukaemia was related to *in vitro* drug resistance. This was determined by analysing the correlation between the S-phase fractions and the *in vitro* resistance to 15 different anticancer agents assessed with the methyl-thiazol–tetrazolium (MTT) assay.

## MATERIALS AND METHODS

### Peripheral blood and bone marrow samples

The local ethical committee has given its permission to perform this study. Bone marrow and peripheral blood samples were collected with informed consent from a total of 452 children with acute leukaemia. From these patients, 362 were diagnosed as ALL and 90 as AML. From the ALL patients, 213 samples were taken at initial diagnosis and 149 at relapsed disease. For the AML samples, 68 were obtained from patients at initial diagnosis and the remaining 22 samples from relapsed patients. In total, 282 bone marrow and 170 peripheral blood samples were included. This cohort of samples has been used for several studies over the past years, but no data have been reported that were focused on drug resistance in relation to cell proliferation. Six different groups participated in the study: the Dutch Childhood Leukaemia Study Group (DCLSG, Den Haag, The Netherlands); the Sophia Children's Hospital, Rotterdam, The Netherlands; the COALL Study Group (Hamburg, Germany); the ALL-BFM Study Group (Berlin, Germany); the AML-BFM Study Group (Münster, Germany); the Rigshospitalet, Copenhagen, Denmark. Immunophenotyping and DNA index flow cytometry were performed following standard procedures at the reference laboratories of the participating groups. Hyperdiploid samples were defined as having a DNA index between 1.16 and 1.35 (Pui *et al*, 1990; Smets *et al*, 1995). B-lineage immunophenotype was defined as HLA-DR^+^/terminal deoxynucleotidyl transferase (TdT)^+^/CD19^+^ ALL and further differentiated as follows: pro-B-ALL (CD10^−^/cytoplasmic *μ* chain (c*μ*)^−^/surface immunoglobulin (sIg)^−^), common (c)-ALL (CD10^+^/c*μ*^−^/sIg^−^) and precursor-B (pre-B)-ALL (CD10^+^ or CD10^−^/c*μ*^+^/sIg^−^). T-lineage (T)-ALL was defined by TdT^+^/cytoplasmic CD3^+^/CD7^+^. Patient characteristics (gender, age, white blood cell count at diagnosis) were collected by study centers.

### Isolation of leukaemia cells

Within 24 h (and in a few cases within 48 h) after sampling, mononuclear cells were separated by density gradient centrifugation at 480 **g** for 15 min (Lymphoprep, 1.077 g ml^−1^, Nycomed Pharma, Oslo, Norway). After washing, the cells were resuspended into culture medium consisting of RPMI 1640 (Dutch modification without L-glutamine; Gibco BRL, Breda, The Netherlands), containing 20% fetal calf serum (FCS; Integro, Zaandam, The Netherlands), 2 mM L-glutamine (ICN Biochemicals, Costa Mesa CA, USA), ITS media supplement (5 *μ*g ml^−1^ insulin, 5 *μ*g ml^−1^ transferrin, 5 ng ml^−1^ sodium selenite) (Sigma, St Louis MO, USA), 80 IU ml^−1^ penicillin (Gibco BRL Breda, The Netherlands) 80 *μ*g ml^−1^ streptomycin (Gibco BRL), 0.1 *μ*g ml^−1^ fungizone (ICN Biomedicals), and 0.2 mg ml^−1^ gentamycin (Flow Laboratories, Irvine, Scotland). Contaminating lymphocytes were removed using immunomagnetic beads as described previously (Kaspers *et al*, 1994). This procedure was carried out at room temperature instead of at 37°C in order to avoid phagocytosis of the beads by the myeloid leukaemia cells. Isolated cells were collected in the culture medium. Only samples that contained more than 80% leukaemic cells, as determined by cytospin preparations stained with May–Grünwald–Giemsa (Merck, Darmstadt, Germany) were included. Not from all samples all parameters could be tested because of limited number of cells and/or failing test(s).

### Methods used for determination of cell proliferation

Only samples that were taken within 24 h after sampling were used for determination of cell proliferation.

### Analysis of S-phase fractions by flow cytometry

Propidium iodide (PI) is a substance that intercalates into double-helical regions of both DNA and RNA molecules. For detection of the DNA content only, isolated leukaemia cells (fixed with 70% ethanol, stored at 4°C) were pretreated with trypsin (T0134, Sigma) for 5 min at room temperature. Subsequently, cells were incubated with ribonuclease A (R4875, Sigma) and trypsin inhibitor (T9253, Sigma) for 10 min at room temperature according to the protocol of Vindelov *et al* (1983a). Fixed cells were stained with PI (Sigma, St Louis MO, USA) for 15 min on ice. Trout red blood cells, which have a DNA content of 80% of the human diploid value, were used as internal reference (Vindelov *et al*, 1983b). The intracellular DNA content was determined by the mean fluorescence intensity of gated leukaemia cells and was expressed as the percentage of all cells in the S phase, which was calculated with the Cell Fit software program for DNA cell cycle analysis (Becton and Dickinson, Mountain View, CA). Since only samples that contained more than 80% leukaemic cells were included, it was assumed that aneuploidy of the leukaemia cells did not interfere with the calculation of the S-phase fraction.

### Flow cytometric detection of proliferation-associated antigens

Ki67 is a nuclear antigen expressed in all phases of the cell cycle, except in the G_0_- and early G_1_-phase. Expression of Ki67 increases with cell cycle progression, and has its maximum expression in late S- and G_2_-phases. Proliferation cell nuclear antigen (PCNA) is the polymerase delta accessory molecule, which plays a role in nucleotide excision repair, and has its maximum concentration at the S- and late G_1_-phase. PCNA is a marker of G_1_-, S-, G_2_- and M-phases of the cell cycle. However, because of the relatively long half-life time, PCNA may also be detected in cells that are in the G_0_-phase.

Isolated leukaemia cells were fixed for 15 min by gently dropping ice-cold methanol on the cell pellet at −20°C. Fixed leukaemia cells were washed and incubated for 45 min at room temperature with monoclonal antibodies against Ki67 antigen (1 : 10) (DAKO, Glostrup, Denmark), PCNA (1 : 100) (DAKO), or control IgG1 (1 : 400) (ITK Diagnostics, Uithoorn, The Netherlands) diluted in phosphate-buffered saline (PBS) containing 1% bovine serum albumine (BSA). Subsequently, cells were washed and incubated for 30 min at room temperature with FITC-conjugated rabbit-anti-mouse F(ab′)2 fragments (1 : 50; DAKO) diluted in PBS containing 1% BSA and 2% pooled human serum. After washing, green fluorescence intensity was detected by flow cytometry through a 530/30 nm bandpass filter set (FACScan; Becton and Dickinson, Erembodegem, Belgium). The expression of the proliferation-associated antigens was determined by the mean fluorescence intensity of gated leukaemia cells and was expressed as the percentage of positively stained cells in relation to the total amount of cells. Positively stained cells were defined as cells that showed higher fluorescence intensity as compared to the highest fluorescence intensity of cells that were incubated with control IgG1 (set as marker).

### *In vitro* drug resistance assay

The MTT assay was performed as described previously (Kaspers *et al*, 1991). In short, isolated leukaemia cells were exposed to six different concentrations of the selected drugs. After 4 days of incubation in 5% CO_2_ humidified air at 37°C, 3-(4,5-dimethylthiazol-2-yl)-2,5-diphenyl tetrazoliumbromide (Sigma, St Louis MO, USA) was added and cells were incubated for another 6 h. Subsequently, formazan crystals formed were dissolved in acidified isopropanol. The optical density, which appeared linearly related to the number of viable cells (Kaspers *et al*, 1991), was measured at 562 nm (Bio-Kinetics Reader, Bio-Tek Instruments, Winooski VT, USA). Samples with less than 70% of leukaemia cells in control wells after 4 days of culture and/or an optical density below 0.050 arbitrary units (adjusted for blank values) were considered not valuable and were excluded from the study. The LC_50_ values, that is, the drug concentration that killed 50% of the cells, were calculated. The *in vitro* drug resistance was determined for the following 15 drugs: 0.008–8.0 *μ*g ml^−1^ aclarubicin (Lundbeck A/S, Copenhagen, Denmark), 0.002–2.5 *μ*g ml^−1^ cytarabine (Pharmacia & Upjohn, Woerden, The Netherlands), 0.002–2 *μ*g ml^−1^ daunorubicin (Rhône Poulenc Rorer, Amstelveen, The Netherlands), 0.002–6 *μ*g ml^−1^ dexamethasone disodiumphosphate (Bufa Pharmaceutical Products, Uitgeest, The Netherlands), 0.008–8 *μ*g ml^−1^ doxorubicin (Pharmacia & Upjohn, Woerdon, The Netherlands), 0.05–50 *μ*g ml^−1^ etoposide (Bristol-Myers Squib, Woerden, The Netherlands), 0.1–100 *μ*g ml^−1^ 4-hydroperoxy ifosfamide, which is the active metabolite of ifosfamide (Asta-Medica, Diemen, The Netherlands), 0.002–2 *μ*g ml^−1^ idarubicin (Pharmacia & Upjohn), 0.003–10 IU L-asparaginase (Christiaens, Breda, The Netherlands), 15.6–500 *μ*g ml^−1^ mercaptopurine (Glaxo Wellcome, Zeist, The Netherlands), 0.001–1 *μ*g ml^−1^ mitoxantrone (AHP Pharma Weyth Lederle, Hoofddorp, The Netherlands), 0.008–2000 *μ*g ml^−1^ prednisolone disodiumphosphate (Bufa Pharmaceutical Products), 0.003–8 *μ*g ml^−1^ teniposide (Bristol-Myers Squib Uitegeest, The Netherlands), 1.56–50 *μ*g ml^−1^ thioguanine (Glaxo Wellcome, Zeist, The Netherlands), and 0.05–50 *μ*g ml^−1^ vincristine (Eli Lilly, Houten, The Netherlands).

### Statistical data analysis

Differences in the distributions of variables between two groups were analysed by the Mann–Whitney *U*-test. In order to identify a relation between two variables within one group, the Spearman rank correlation coefficients were calculated. If less than nine samples were available, however, this was considered as too small a number for meaningful analysis. All analyses were two-tailed and differences were considered statistically significant when *P*⩽0.05. The samples taken at initial diagnoses and the samples taken at relapse are largely unmatched, that is, from different individuals.

## RESULTS

### Cell proliferation

#### Peripheral blood vs bone marrow samples

**Figure 1:**
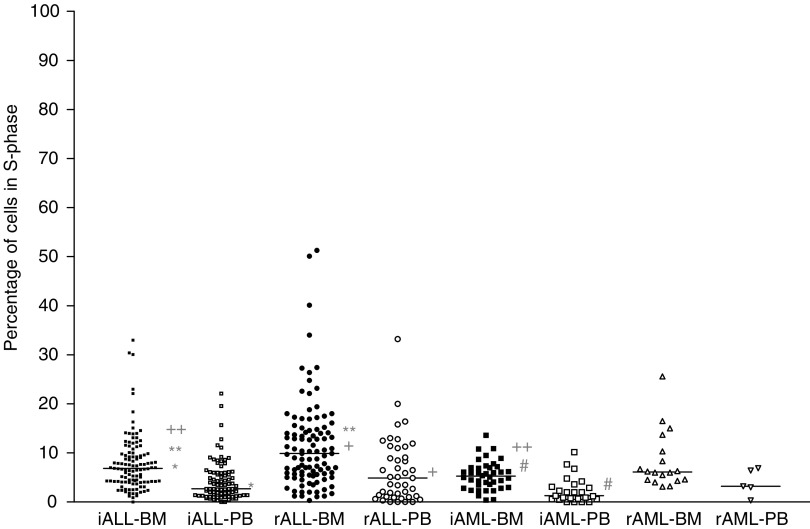
Comparison of S-phase fractions of peripheral blood (PB) *vs* bone marrow (BM) samples. Represented are the percentages of cells in S phase for the ALL and AML samples derived from BM or PB. The median value is shown as horizontal bar. Both the initial and relapse ALL samples as well as the initial AML samples derived from BM had a significantly higher S-phase fraction than those derived from PB (^*^, ^+^, ^#^; *P*<0.01). The BM-derived ALL samples taken at initial diagnosis differed significantly from those taken at relapsed disease in the S-phase fraction (^**^; *P*<0.01). In addition, the BM-derived ALL samples taken at initial diagnosis differed significantly from the initial AML samples (^++^; *P*<0.05). iALL and iAML, samples taken at initial diagnosis; rALL and rAML, samples taken at relapsed disease.

**Figure 2:**
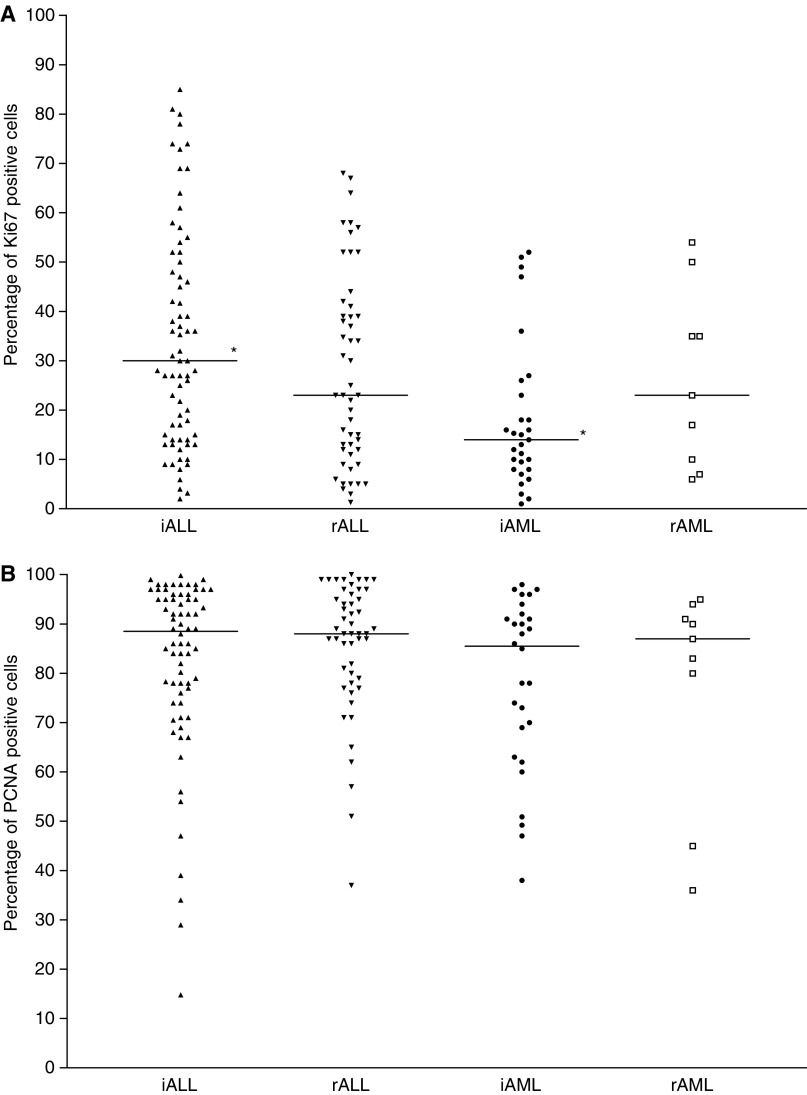
(**A**) Represented are the percentages of Ki67-positive cells for the different childhood acute leukaemia subgroups. The median value is shown as horizontal bar. The ALL samples taken at initial diagnosis differed significantly from the AML samples taken at initial diagnosis in Ki67 expression (^*^; *P*<0.05). iALL and iAML, samples taken at initial diagnosis; rALL and rAML, samples taken at relapsed disease. (**B**) Represented are the percentages of PCNA-positive cells for the different childhood acute leukaemia subgroups. The median value is shown as horizontal bar. No significant differences in PCNA expression in the different subgroups were observed. iALL and iAML, samples taken at initial diagnosis; rALL and rAML, samples taken at relapsed disease.

In both ALL and AML samples taken at initial diagnosis, the S-phase fraction was higher in bone marrow than in peripheral blood samples (in initial ALL, median values resp. 6.9 and 2.7%; in initial AML resp. 5.3 and 1.3%; both *P*<0.01) (Figure 1

). Similarly, in ALL samples, but not in AML samples, taken at relapse more leukaemia cells in S-phase were detected in bone marrow as compared with peripheral blood samples (in relapsed ALL, median values resp. 9.9 and 4.9%; *P*<0.01) (Figure 1). However, no significant differences in Ki67 or PCNA expression were found between bone marrow and peripheral blood samples. In general, the expression of Ki67 antigen varied greatly from patient to patient, whereas the expression of PCNA was generally high in all patients (Figure 2

). Since the S-phase fraction differed between the bone marrow and peripheral blood samples, the cell proliferation data were analysed separately for both types of samples. It was decided to further limit the analysis of the cell proliferation data to bone marrow as the site of origin of leukaemia and the main location of minimal residual disease and initial site of relapses. Data on cell proliferation are illustrated in Figure 1 and Figure 2. Taking all bone marrow samples together irrespective of disease, the S-phase correlated both with Ki67 (*n*=155, *ρ*=0.32, *P*<0.01) and PCNA expression (*n*=152, *ρ*=0.24, *P*<0.05), and the Ki67 expression correlated with the PCNA expression (*n*=176, *ρ*=0.352, *P*<0.01).

### Initial *vs* relapsed acute leukaemia

In ALL bone marrow samples from relapsed patients an increased number of cells in S phase was detected when compared with ALL bone marrow samples from initial patients (median values, resp. 9.9 and 6.9%; *P*<0.01). However, no significant differences in Ki67 and PCNA expression were detected. No differences in cell proliferation parameters were found between initial and relapsed AML. Within the initial ALL samples, the S-phase fractions correlated with both Ki67 (*n*=62, *ρ*=0.37; *P*<0.01) and PCNA expression (*n*=61, *ρ*=0.30; *P*<0.05). In addition, Ki67 expression was related to PCNA expression (*n*=70, *ρ*=0.46; *P*<0.01). In the relapse ALL samples, Ki67 expression was related to the S-phase fraction (*n*=44, *ρ*=0.34, *P*<0.05). Within all AML samples, the S-phase fraction was related to Ki67 expression (*n*=34, *ρ*=0.38, *P*<0.05). However, there was discrepancy between the S-phase fraction and the expression of Ki67 and PCNA in both the initial AML and relapse AML samples, probably because of the low number of samples tested (maximum number of samples tested resp. *n*=28 and 9). Data are illustrated in Figure 1 and Figure 2.

### Acute lymphoblastic *vs* myeloblastic leukaemia

A higher number of cells in S-phase were detected in initial ALL bone marrow samples compared to initial AML bone marrow samples (median value of 6.9% in ALL and 5.3% in AML; *P*<0.05). In addition, a higher expression of Ki67 was found in initial ALL samples when compared with initial AML samples (median value of 30% in ALL and 14% in AML; *P*<0.05). No significant difference between ALL and AML samples was found in the PCNA expression. Comparing the relapse ALL samples with the relapse AML samples, no significant differences were found in the various cell proliferation features. Data are illustrated in Figure 1 and Figure 2.

### *In vitro* drug resistance

In correspondence with our previous data, no differences were found in resistance to any of the drugs between bone marrow and peripheral blood samples (Kaspers *et al*, 1991; Klumper *et al*, 1995a). For this reason, the drug resistance data of bone marrow and peripheral blood samples were analysed all together. Drug resistance data obtained in the present study are largely in agreement with previously published findings of our group (Klumper *et al*, 1995a,1995b; Zwaan *et al*, 2000). Summarising, leukaemia cells isolated from ALL patients were significantly more sensitive than those derived from AML patients to 12 out of the 15 drugs: prednisolone, dexamethasone, L-asparaginase, vincristine, daunorubicin, doxorubicin, idarubicin, aclarubicin, mitoxantrone, etoposide, teniposide, and 4-hydroperoxy ifosfamide. The ALL samples taken at initial diagnosis were more sensitive compared with relapse ALL samples to eight out of the 15 drugs: prednisolone, dexamethasone, L-asparaginase, vincristine, daunorubicin, doxorubicin, idarubicin, and thioguanine. For the AML samples, the *in vitro* drug resistance did not differ significantly between initial and relapse samples, but the numbers were small in the latter group.

### Correlation between S-phase fractions and *in vitro* drug resistance

The S-phase fractions, but not the expression of Ki67 or PCNA, showed differences between bone marrow *vs* peripheral blood samples as well as between initial *vs* relapsed ALL. For this reason, it was decided to restrict the correlation data of *in vitro* drug resistance to S-phase fractions only. However, essentially similar results were found for the other proliferation parameters. All significant correlations that were found between S-phase fractions and drug resistance were inverse, that is, higher S-phase fractions were related to lower LC_50_ values or a more drug-sensitive phenotype. In ALL samples taken at initial diagnosis increased S-phase fractions correlated with sensitivity to cytarabine, L-asparaginase, mercaptopurine, teniposide, thioguanine, and vincristine (Table 1

**Table 1:** Represented are the Spearman rank correlation coefficients (*ρ*) between cell proliferation features and drug resistance in bone marrow-derived childhood acute leukaemia samples

	**Relation with S-phase (*ρ*)**			
**Anticancer agents**	**iALL (*n*)**	**rALL (*n*)**	**iAML (*n*)**	**rAML (*n*)**
Aclarubicin	−0.05 (25)	−0.05 (24)	−0.49 (10)	—
Cytarabine	−0.30* (53)	−0.07 (29)	−0.15 (29)	0.39 (9)
Daunorubicin	−0.23 (60)	−0.29 (27)	−0.09 (31)	0.23 (11)
Dexamethasone	−0.20 (56)	0.18 (13)	−0.67* (12)	—
Doxorubicin	−0.19 (62)	−0.24 (25)	0.05 (29)	0.27 (11)
Etoposide	—	—	−0.08 (30)	0.56 (11)
Ifosfamide	0.19 (41)	0.07 (24)	−0.15 (13)	—
Idarubicin	−0.03 (28)	−0.28 (25)	−0.33 (17)	—
L-asparaginase	−0.37** (62)	0.27 (24)	−0.24 (22)	0.06 (8)
Mercaptopurine	−0.39** (58)	—	−0.39 (9)	—
Mitoxantrone	−0.21 (55)	−0.31 (24)	−0.32 (21)	—
Prednisolone	−0.14 (58)	0.00 (24)	−0.18 (31)	0.26 (10)
Teniposide	−0.32* (50)	−0.08 (18)	−0.26 (11)	—
Thioguanine	−0.30* (57)	0.01 (29)	−0.27 (29)	0.32 (10)
Vincristine	−0.26* (63)	−0.21 (27)	−0.28 (31)	0.31 (11)

iALL and iAML, samples taken at initial diagnosis; rALL and rAML, samples taken at relapsed disease; *n*, number of samples; significant difference at level

* *P*<0.05;

** *P*<0.01.

). For the ALL samples taken at relapsed disease, the S-phase fractions did not correlate with sensitivity to any of the tested drugs (Table 1). Within AML samples taken at initial diagnosis, a correlation was found between the S-phase fraction and sensitivity to dexamethasone (Table 1). Although it has to be mentioned that most AML samples tested were extremely resistant for dexamethasone and the number of tested AML samples for this particular drug was small (*n*=12). No relations were found between the S-phase fractions and *in vitro* drug resistance in AML samples taken at relapsed disease, although the number of samples in this group was rather small (Table 1).

### Correlation between S-phase fractions and *in vitro* drug resistance within subgroups of initial ALL

Within the ALL bone marrow samples taken at initial diagnosis, the S phase was not related to white blood cell count, age, or gender. Within the initial ALL samples, the hyperdiploid samples (*n*=31), defined as having a DNA index between 1.16 and 1.35 (Pui *et al*, 1990; Smets *et al*, 1995), had a significantly higher S-phase fraction (median value 7.9%) compared to nonhyperdiploid samples (*n*=83; median S-phase fraction of 6.3%; *P*=0.04). Within this hyperdiploid subgroup no correlations were found between S phase and *in vitro* resistance to any of the tested drugs, probably because of the small numbers of samples that were tested for both parameters (maximum of *n*=18). Within the nonhyperdiploid subgroup, a higher S phase was significantly related to increased sensitivity to cytarabine (*ρ*=−0.35; *P*=0.02), L-asparaginase (*ρ*=−0.36; *P*=0.02), mercaptopurine (*ρ*=−0.44; *P*=0.003), mitoxantrone (*ρ*=−0.31; *P*=0.05), teniposide (*ρ*=−0.39; *P*=0.01), and thioguanine (*ρ*=−0.39; *P*=0.01) (maximum of *n*=45 with both parameters available). Within the initial ALL samples, the two major immunophenotypic subgroups, that is, common/precursor-B-lineage (c/pre-B-)ALL, and T-lineage (T-)ALL differed significantly (*P*=0.02) in S-phase fractions (S-phase fraction of 6.7% (*n*=83) and 11.3%, respectively (*n*=11). Within the c/pre-B-ALL, increased sensitivity to both L-asparaginase (*ρ*=−0.32; *P*=0.02) and mercaptopurine (*ρ*=−0.39; *P*=0.01) was significantly related to a higher S-phase fraction (maximum of *n*=51 with both parameters available). Within the T-ALL subgroup, the number of samples analysed for both parameters was too small for analysis (*n*=5).

## DISCUSSION

In the present study, cell proliferation of different subgroups of childhood acute leukaemia was defined and correlated with *in vitro* drug resistance. Bone marrow samples had generally a higher S-phase fraction than blood samples, which is in correspondence with previous findings of Dow *et al* (1982). The high proliferative activity of bone marrow leukaemia cells might be caused by stimulation with growth factor-producing stromal cells that are present in the patient's bone marrow. This is strengthened by the fact that at the end of the *in vitro* drug assay, after 4 days of culture without growth factors, no significant differences were detected anymore in the S-phase fractions between the leukaemia cells derived from bone marrow and blood (data not shown).

The S-phase fraction (but not Ki67 and PCNA expression) of relapse ALL samples was significantly higher than that of initial ALL samples. In addition, both the S-phase fraction and Ki67 expression (but not PCNA expression) were significantly higher in childhood ALL than in childhood AML samples, which is in agreement with other findings (Ito *et al*, 1992). The expression of Ki67 antigen differed greatly from patient to patient whereas the expression of PCNA was generally high in all acute leukaemia patients, which is in accordance with previous reports (Ito *et al*, 1992; Tsurusawa *et al*, 1992). The high percentage of cells that were positive for PCNA in all leukaemia subgroups can be explained by the long half-life time of this antigen. In conclusion, PCNA and Ki67 appeared to be less attractive as proliferation markers than the S-phase fraction, since these markers were not able to differentiate the various subgroups of childhood acute leukaemia.

*In vitro* drug resistance, assessed with the MTT assay, is significantly related to the clinical response to chemotherapy in childhood ALL (Klumper *et al*, 1995b; Hongo *et al*, 1997; Kaspers *et al*, 1997,^1998^; Asselin *et al*, 1999). In addition, in childhood AML clinical outcome and *in vitro* drug resistance showed to correlate as well (Klumper *et al*, 1995a). In the present study an increased *in vitro* sensitivity to antimetabolites (cytarabine, mercaptopurine, thioguanine), L-asparaginase, vincristine, and teniposide was related to a higher S-phase fraction of leukaemia cells isolated from initial childhood ALL patients. In addition, the other proliferative markers (Ki67 and PCNA) were related to the antimetabolites, vincristine, teniposide as well as for the anthracyclins (data not shown). It is understandable that the antimetabolites were related to cell proliferation since these drugs inhibit DNA and RNA synthesis, and are therefore especially effective for cells in S-phase. A relation between high S-phase fraction of leukaemia cells and an increased sensitivity to vincristine or L-asparaginase has not been described before in the literature. Additionally, we found a relation between an increased sensitivity to the epipodophyllotoxin, teniposide, and a higher S-phase fraction. Epipodophyllotoxins are inhibitors of topoisomerase-II-alpha that are expressed in a cell cycle-dependent manner (Kellner *et al*, 2000). We did not find, however, a relation between the S-phase fraction and sensitivity to anthracyclines that are also known to inhibit topoisomerase-II, although anthracyclins share other action mechanisms also.

Within the initial ALL samples, a correlation between a higher S-phase fraction and an increased sensitivity to various drugs was also observed in the nonhyperdiploid subgroup (cytarabine, L-asparaginase, mercaptopurine, mitoxantrone, teniposide, and thioguanine) as well as in the c/pre-B ALL subgroup (L-asparaginase, mercaptopurine). Moreover, within the initial ALL samples, the S-phase fraction was not related to white blood cell count, age, or gender. These findings further supports the idea that there is a direct relation between cell proliferation and drug sensitivity in childhood ALL at initial diagnosis and that the observed relation not merely reflects a difference in both cell proliferation and *in vitro* drug sensitivity in the various ALL subgroups. Both in relapse ALL samples and in AML samples, no such relation was found between S-phase fraction and *in vitro* drug resistance as in the initial ALL samples.

Relapse ALL samples had an increased S-phase fraction and they were more resistant to various drugs than intial ALL samples. Apparently, not only the proliferative status of leukaemia cells accounts for its primary response to a particular drug. Especially for leukaemia cells from relapsed disease, drug-resistant clones may have emerged and for these cells intrinsic cellular resistance probably plays a more important role in the response to drugs than the proliferative status.

An increased proliferative activity may increase the sensitivity to particular drugs, therefore pretreatment with growth factors prior to treatment with these proliferation-dependent drugs may increase the response rate in childhood acute leukaemia. Several studies have indeed demonstrated that the *in vitro* sensitivity to cytarabine of AML cells could be enhanced by preincubation with granulocyte colony-stimulating factor (G-CSF) and/or with granulocyte–macrophage colony-stimulating factor (GM-CSF) (Butturini *et al*, 1990; Bai *et al*, 1999). Clinically, pretreatment with G-CSF (as in the FLAG regimen, which is a combination therapy of fludarabine, cytarabine, and G-CSF), seems to be effective and well tolerated in the treatment of poor-risk AML patients (Montillo *et al*, 1998; Jackson *et al*, 2001). The efficacy of this therapy, however, has not yet been determined in a controlled, randomised clinical study. In the present study, especially the S-phase fractions of untreated childhood ALL samples appeared to correlate with the *in vitro* sensitivity to a number of anticancer agents. In childhood ALL patients pretreatment with growth factors has not been adequately investigated as a chemosensitising approach, but the present data do suggest that this approach may be useful.
